# Intelligent Classification of Urban Noise Sources Using TinyML: Towards Efficient Noise Management in Smart Cities

**DOI:** 10.3390/s25206361

**Published:** 2025-10-14

**Authors:** Maykol Sneyder Remolina Soto, Brian Amaya Guzmán, Pedro Antonio Aya-Parra, Oscar J. Perdomo, Mauricio Becerra-Fernandez, Jefferson Sarmiento-Rojas

**Affiliations:** 1 School of Science and Engineering, Universidad del Rosario, Bogotá 111711, Colombia; 2 School of Medicine and Health Sciences, Universidad del Rosario, Bogotá 111711, Colombia; 3 Department of Electrical and Electronic, Universidad Nacional de Colombia, Bogotá 111321, Colombia

**Keywords:** urban noise, public health, TinyML, acoustic models, ML classification, YAMNet

## Abstract

**Highlights:**

**What are the main findings?**

- An efficient TinyML system was deployed for real-time, on-device classification of urban noise, achieving high accuracy (precision/recall up to 1.00).
- Heavy vehicles were identified as the most frequent noise sources, whereas aircraft generated the highest A-weighted sound pressure levels (L_a_, max = 88.4 dB(A)), exceeding the local permissible limit by up to 18 dB(A).

**What are the implications of the main findings?**

- The study proves that TinyML is a viable solution for dense, permanent noise monitoring networks that identify specific sources, not just volume.
- It enables targeted noise mitigation policies by pinpointing the most impactful contributors to urban noise pollution.

**Abstract:**

Urban noise levels that exceed the World Health Organization (WHO) recommendations have become a growing concern due to their adverse effects on public health. In Bogotá, Colombia, studies by the District Department of Environment (SDA) indicate that 11.8% of the population is exposed to noise levels above the WHO limits. This research aims to identify and categorize environmental noise sources in real time using an embedded intelligent system. A total of 657 labeled audio clips were collected across eight classes and processed using a 60/20/20 train–validation–test split, ensuring that audio segments from the same continuous recording were not mixed across subsets. The system was implemented on a Raspberry Pi 2W equipped with a UMIK-1 microphone and powered by a 90 W solar panel with a 12 V battery, enabling autonomous operation. The TinyML-based model achieved precision and recall values between 0.92 and 1.00, demonstrating high performance under real urban conditions. Heavy vehicles and motorcycles accounted for the largest proportion of classified samples. Although airplane-related events were less frequent, they reached maximum sound levels of up to 88.4 dB(A), exceeding the applicable local limit of 70 dB(A) by approximately 18 dB(A) rather than by percentage. In conclusion, the results demonstrate that on-device TinyML classification is a feasible and effective strategy for urban noise monitoring. Local inference reduces latency, bandwidth usage, and privacy risks by eliminating the need to transmit raw audio to external servers. This approach provides a scalable and sustainable foundation for noise management in smart cities and supports evidence-based public policies aimed at improving urban well-being. This work presents an introductory and exploratory study on the application of TinyML for acoustic environmental monitoring, aiming to evaluate its feasibility and potential for large-scale implementation.

## 1. Introduction

Sound is the perception or sensation produced in the human ear by the vibration of acoustic mechanical waves, which are transmitted through an elastic medium, such as air. When this perception or sensation is experienced as an annoying, unpleasant, or untimely sound for the person perceiving it, it can be classified as noise. Depending on its nature and emission levels, noise can cause environmental harm, referred to as acoustic pollution in spaces where it is evident, for example, in industrial, commercial, recreational, or everyday human activities such as shouting, parties, or vehicle use [1].

The World Health Organization (WHO) has identified environmental noise as one of the most harmful factors to human health. According to a study, at least one million healthy life years are lost each year due to traffic-related noise in Western Europe [2]. This figure, expressed in Disability-Adjusted Life Years (DALY), represents the total number of healthy years lost due to illness, disability, or premature death caused by noise exposure. This study reveals alarming figures about the disease burden caused by environmental noise. It is estimated that approximately 61,000 years are lost annually due to ischemic heart disease in Western European countries. Additionally, cognitive impairment in children aged 7 to 19 represents a loss of around 45,000 years in the same region. Noise-induced sleep disturbance affects residents of European Union cities with more than 50,000 inhabitants, accounting for nearly 903,000 lost healthy life years annually. Noise-induced tinnitus causes a loss of approximately 22,000 years in the adult population of Western Europe. Finally, annoyance caused by noise results in the loss of nearly 587,000 years in the urban population of the European Union. These data highlight the significant impact of environmental noise on the population’s health and well-being, emphasizing the urgent need for policies and strategies to mitigate these negative effects.

Within the framework of smart cities, efficient management of urban noise has become a priority for improving residents’ quality of life. Smart cities employ advanced monitoring technologies to identify and mitigate sources of acoustic pollution, enabling continuous tracking of noise levels and real-time data analysis for informed decision-making [3]. For example, the implementation of acoustic sensors and Big Data analysis can help identify noise hotspots and develop specific strategies to reduce citizens’ exposure to excessive noise. Additionally, integrating innovative solutions such as smart acoustic barriers and urban planning that promotes quiet zones is essential for addressing this issue within the framework of smart cities.

In the Colombian context, the Ministry of Environment and Sustainable Development, through the Urban Environmental Quality Index (ICAU), establishes the indicator Percentage of Urban Population Exposed to Environmental Noise above a reference value (PUAR) to assess the impact of acoustic pollution on people [4]. This indicator allows for comparison between different cities regarding exposure and potential exposure to high noise levels through a percentage-based classification.

According to Iqbal H. Sarker, machine learning (ML) is a subfield of artificial intelligence focused on the development of algorithms that enable computers to learn and make predictions based on data [5]. In this context, the implementation of machine learning in sound classification has been essential, using robust algorithms such as neural networks and decision trees to identify patterns in audio data from spectrograms and Mel Frequency Cepstral Coefficients (MFCC). In practical applications, ML is used for anomaly detection in security systems, music genre classification, and emotion recognition in voice [6,7].

Sound classification is a crucial task in the field of smart cities, including acoustic surveillance, biodiversity monitoring, anomaly detection in machines, and human–machine interaction. To address this task, various Artificial Intelligence (AI) techniques, Genetic Algorithms [8], and machine learning (ML), along with some of its derivatives, such as Tiny Machine Learning (TinyML) [9], have been used, which can be applied on edge devices.

Specifically, TinyML refers to the implementation and execution of machine learning models on low-power microcontrollers or edge devices such as microprocessors, cell phones, tablets, and others that are typically used as Internet of Things (IoT) objects. These embedded systems are highly energy-efficient and capable of operating in environments with very limited resources, such as memory and processing capacity.

TinyML is revolutionizing sound classification on resource-constrained devices, edge devices, and microcontrollers [10]. TinyML techniques can perform real-time audio classifications tasks using algorithms optimized to consume very little power and memory. This is crucial for IoT applications where devices need to operate efficiently and autonomously for extended periods [11].

Unlike cloud-based processing, on-device TinyML avoids the need to transmit large audio files continuously, reducing bandwidth and infrastructure costs. It also enables real-time responses in areas with limited or intermittent connectivity and ensures greater privacy by keeping raw audio data on the device.

Processing data directly on the IoT device minimizes latency, bandwidth usage, and infrastructure costs, while enhancing privacy and scalability for smart-city noise monitoring. This edge-based approach also enhances privacy and enables scalable deployments for smart city noise monitoring. The combination of advanced technologies such as miniature machine learning offers new opportunities to effectively address acoustic pollution in urban environments. These solutions not only enable more efficient real-time noise management but also facilitate the implementation of preventive strategies in smart cities. In this way, they contribute to the well-being of the population by minimizing the negative impacts of environmental noise on health and quality of life. It should be noted that this research represents an introductory and exploratory effort to assess the feasibility and performance of TinyML models for environmental acoustic monitoring under real-world conditions.

## 2. Related Work

In recent years, environmental sound classification has gained renewed attention due to advances in technology and the growing demand for accurate sound detection in dynamic environments. A leading author in this field is Karol Picza [12], who delves into the potential of convolutional neural networks (CNNs) for sound classification in his work. Unlike traditional techniques that rely on manually defined audio features, CNNs can learn directly from the data without prior intervention, which can lead to more accurate and robust classification [13].

This idea arises from the proposal by Justin Salamon, Christopher Jacoby, and Juan Pablo Bello, who argue for the importance of having specific datasets and taxonomies, such as those focused on urban sounds. By creating the UrbanSound 8k database, a structured and diverse dataset can be key to training models that generalize well in real-world urban conditions [14].

Building on these advances, subsequent studies have explored innovative deep-learning architectures to further improve classification performance. Yu Su, Ke Zang, Jingyu Wang, and Kurosh Madani used a neural network to combine the best features of multiple architectures to achieve optimal classification for environmental sound classification [15].

Ahmmed et al. [15] proposed a hybrid neural architecture that combines the most effective features of multiple models. Mesaros et al. [16] introduced the Mixup data augmentation technique that creates training examples by interpolating between pairs of examples and their labels, which can lead to more robust models that are less prone to overfitting [16]. Meanwhile, Anna María Mesaros, Toni Heittola, and Tuomas Virtanen researched the importance of datasets in research. By presenting a dataset specifically for acoustic scene classification, they advocated for the creation of more specialized resources to address specific challenges in audio classification [17].

Audio classification, particularly of environmental and urban sounds, has rapidly evolved due to advances in deep learning and the availability of specialized datasets. As these techniques continue to develop, broader and more sophisticated applications in automatic sound detection and acoustic scene classification are likely to emerge, spanning various contexts from urban environments to security and health applications.

This work focused on the development and implementation of a system for classifying sources of urban noise pollution in Bogotá, Colombia. Using Machine Learning (ML) strategies implemented in TinyML embedded systems, or to be integrated into acoustic pollution monitoring stations, the system enables real-time classification of various noise sources in a specific acoustic environment.

This study advances the field of urban sound classification and encourages reflection on strategies to improve the acoustic environment of cities. Open questions are raised about how these findings can be applied in other metropolises facing similar challenges, highlighting the relevance of the study and the need for more advanced tools for effective urban noise management.

## 3. Materials and Methods

**Figure 1:**
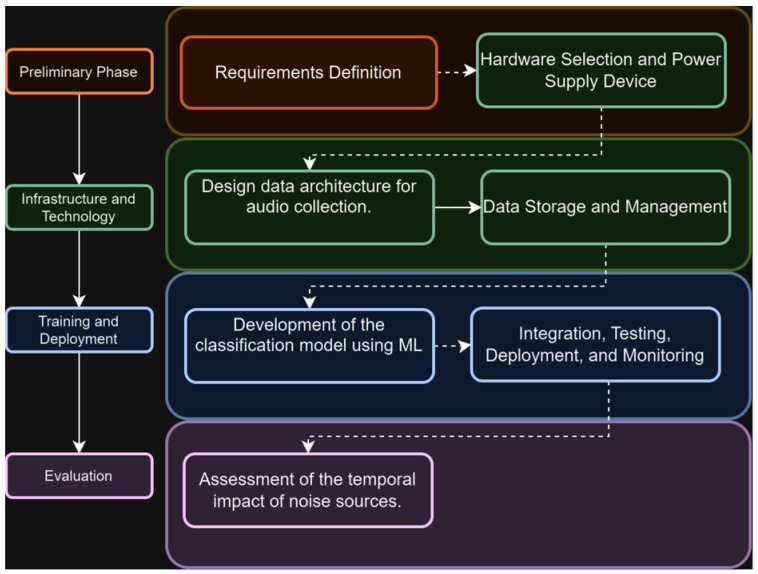
Methodology and Phases of Research.

The project was structured into four phases. As shown in Figure 1, Phase I focused on defining requirements and configuring the hardware. Phase II, infrastructure and technology, centered on data collection as well as the design of the data architecture, including its storage and management. Phase III involved the development of the classification model and its integration with Tiny Machine Learning to solve the problem. Finally, Phase IV included the evaluation of results.

### 3.1. Preliminary Phase

Before project implementation, the selection of hardware and the design of the power system were detailed. Raspberry Pi 2W (Raspberry Pi Ltd., Cambridge, UK) was chosen for its advantages in processing power and memory over Raspberry Pi 3. The Umik-1 microphone (miniDSP Ltd., Hong Kong, China) was selected for its efficiency in capturing high-quality audio. Additionally, a PS 90 solar panel (Photonic Solar Ltd., Bogotá, Colombia), ensures a reliable energy supply. The firmware has been optimized to ensure efficient real-time data capture and processing.

Among several low-power IoT boards ESP32 (Espressif Systems, Shanghai, China), Arduino Nano BLE (Arduino LLC, Somerville, MA, USA), Jetson Nano (NVIDIA Corporation, Santa Clara, CA, USA), the Raspberry Pi 2W was selected for its balanced trade-off between processing capacity, RAM, audio interface compatibility, community support, and low energy use—key factors for real-time TinyML inference in urban environments.

#### 3.1.1. Requirements Definition

Functional requirements define the components that the system will be able to analyze and classify. As the project progresses, these requirements transform into inputs, models, algorithms, and much of the system’s functionality. In agile methodologies, these functionalities are expressed in terms of user stories, using the language that the user would employ. This approach focuses attention on their needs and issues, and on how what is being built meets their expectations [18]. Thus, the following functional requirements were established for the development of the device.

Unlike functional requirements, which focus on the specific functionalities of the system, non-functional requirements focus on key aspects such as usability, interoperability, portability, security, performance, and high availability that the application must have. These requirements are presented in Table 1.

#### 3.1.2. Hardware and Power Supply Device Selection

Based on the requirements and technologies implemented in the market for measuring acoustic indicators, microphones such as the MTI M2211 microphone (MTI Instruments Inc., Albany, NY, USA) are used. Table 2 presents three microphones with prices lower than the estimated value, the SPH0645 microphone (Adafruit Industries, New York, NY, USA), Sunfounder Raspberry and Umik-1 (miniDSP Ltd., Hong Kong, China) that can capture audio with the necessary fidelity. The tests were carried out by recording audio for a day to know the behavior of the microphone, based on the frequency range, sensitivity, audio quality and weather protection.

**Figure 2:**
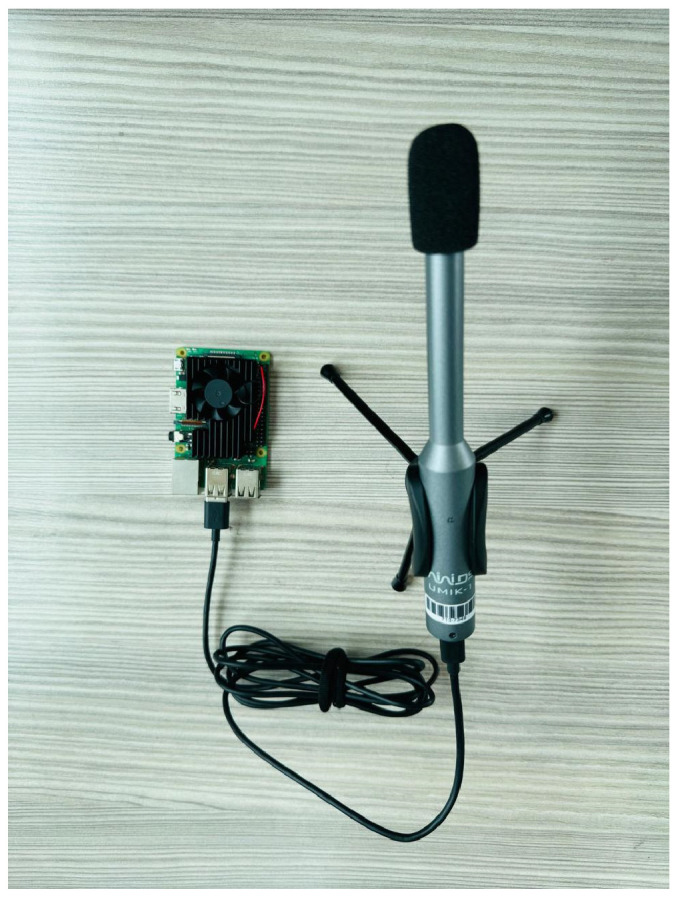
Microphone Umik-1.

- **Microphone:** Based on the results presented, the most cost-efficient microphone was the Umik-1, see Figure 2, whose audio does not suffer high distortion with recordings for long periods of time. However, it was necessary to improve weather protection and filter the audio for the machine learning model.

- **Development board:** The development board [22] has among its significant advantages its compact design and low power consumption compared to other Raspberry Pi models. The Raspberry Pi 2W offers 512 MB of RAM, which is sufficient for lightweight applications such as basic real-time data collection, simple IoT projects, or educational use. In addition, it integrates a quad-core ARM Cortex-A53 processor, enabling efficient processing while maintaining reduced energy requirements. The Raspberry Pi 2W also relies on microSD cards for storage, providing flexibility in terms of capacity and ease of system updates. These characteristics make it an attractive option for projects that require a balance between performance, portability, and energy efficiency at a low cost.
- ***System structure:*** In the energy section of the project, a PS 90 solar panel with a nominal capacity of 90 W [23] has been incorporated. This panel can generate a maximum current of 4.33 A at an operating voltage of 18.625 V, making it suitable for small to medium-scale systems. The panel’s open-circuit voltage is 21.96 V, and the short-circuit current is 4.69 A, providing a safety margin and efficiency during fluctuations in environmental conditions. Weighing 7.2 kg and with dimensions of 905 × 673 × 35 mm, this panel is manageable and fits well in installations with limited space or specific mounting requirements.

**Figure 3:**
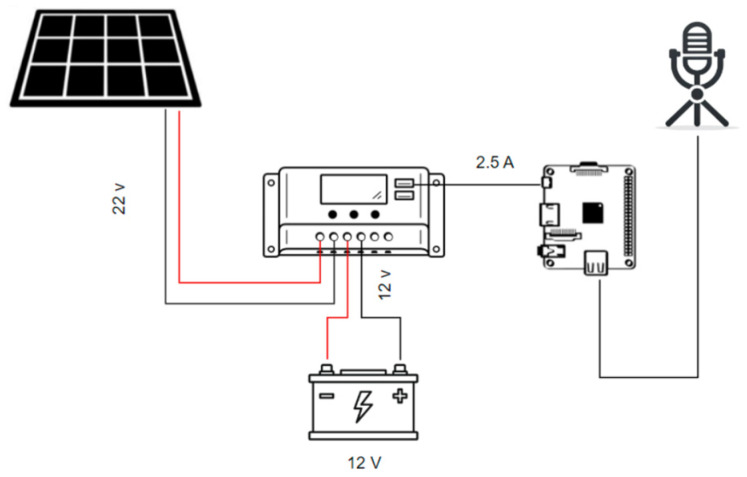
General schematic of the data acquisition system.

Figure 3 shows the integration of this panel with a 40-amp solar PWM charge controller and a 12-volt battery, forming a robust and reliable power system. The controller not only optimizes the charging and discharging of the battery but also protects against overcharging and deep discharge, which is crucial for maintaining the battery’s health over the long term. The integrated USB ports on the controller facilitate direct charging of the Raspberry Pi 2w, making this system a comprehensive, efficient, and adaptive energy solution, ideal for remote areas or as a backup power source in emergencies.

The system’s average power consumption was measured at approximately 2.5 W in continuous operation (equivalent to about 60 Wh per day). Under typical solar conditions in Bogotá, the 90 W photovoltaic panel produces 270–315 Wh daily, assuming 30% system losses. This provides a daily energy margin exceeding 200 Wh, ensuring continuous operation.

Alternatively, the development of software that operates directly on the IoT device for sound data collection (known as firmware), was optimized to handle data collection, pre-processing and transmission efficiently. Firmware robustness is essential for system reliability under variable conditions and over extended periods of operation. Therefore, Programming was implemented in C/C++ (version 11) and MicroPython (version 1.19.1) to enable efficient real-time capture and signal processing algorithms to filter noise and normalize the audio.

#### 3.1.3. Infrastructure, Technology, and Data Architecture for Audio Collection

The workflow designed and implemented for audio collection is presented below, with the aim of applying transfer learning techniques and obtaining a new model [24]. The algorithm first imported the required audio-processing libraries. It then created folders to store the recorded sounds, organized by class: motorcycles, alarms, heavy vehicles, applause, environment, humans, airplanes, and impacts.

**Figure 4:**
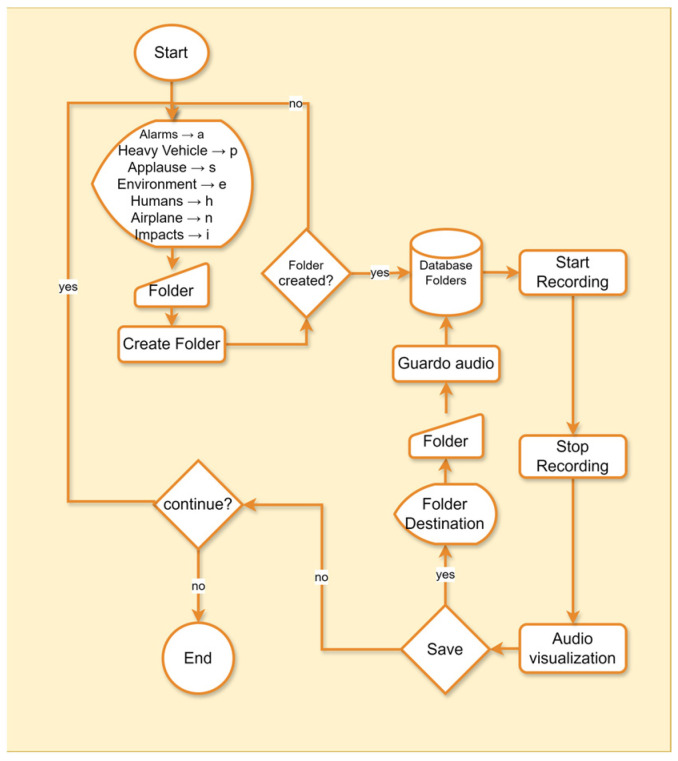
Data collection algorithm.

The recording process was initiated through a menu option, allowing the user to start and stop audio capture. During the recording, each audio file was categorized based on the presence of a single sound source and stored in the folder designated for its class, using the keys assigned to each category. Finally, the user stopped the recording, completing the audio collection cycle or database, as shown in Figure 4.

#### 3.1.4. Data Storage and Management

An unprocessed audio file requires 5.44 MB of storage per minute and 7.833 GB for the 1440 min corresponding to one (1) day. Therefore, to ensure the system operates autonomously for a period of 7 days, at least 54.835 MB of free memory space is required. Due to energy consumption and portability, a 64 GB MicroSD was selected to store audio recordings for 24 h over a period of 8 days.

For audio storage, the processing of the sample collection code and the importance of recording audio every minute, synchronized with the timestamp from the Environmental Noise Monitoring Network of Bogotá (RMRAB) [25], were considered. Therefore, it was necessary to allow a time lapse for processing the storage of audio in WAV format and tracking information in a CSV file. As a result, the audio recording process was conducted for a duration of 59.48 s, with 0.52 s allocated for storage processing, and the next minute of recording began at the 00 s mark of each minute. It is important to clarify that the last second of analysis could be reduced to 0.48 s, according to YAMNet’s official documentation, which sets this time interval as the minimum necessary for extracting classification information see Table 3.

### 3.2. Dataset Preparation and Audio Processing

The classification model plays a key role in the entire process of data collection and analysis. Based on this, Table 4 presents the classes for audio collection, their definitions, and their relevance in daily life.

For this process, the audios were first categorized, and relevant audio folders were selected. Folder conditions and audio lists for each type were established, processing each audio through pre-filtering, amplification, and resampling. The audios were then divided into training, validation, and test sets. Finally, the codes were stored in .CSV files and organized into training folders.

#### 3.2.1. Model Re-Training

**Figure 5:**
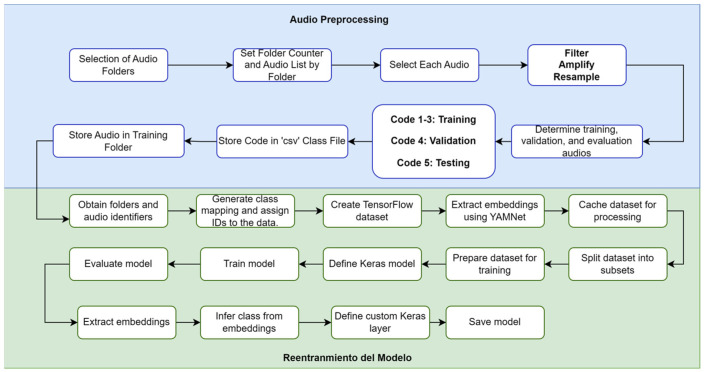
Model re-training algorithm.

The folders previously identified with useful audios were obtained to generate class assignments and create a Tensor Flow dataset compatible with the YAMNet library. As Presented in Figure 5, embeddings, which refer to a natural language processing technique that converts human language, or in this case sounds, into mathematical vectors were extracted using YAMNet and caught for processing. The dataset is then split using train-test split to obtain the training, validation and test sets. From there, the data is prepared and defined in a Keras model, to train and evaluate the model, the embeddings are extracted, and class inferences are generated from them. Finally, the custom Keras layers are defined, and the resulting model is stored see Figure 5.

In addition, during the creation of the five subsets for each class, it was ensured that audio segments originating from the same continuous recording were not split across the training, validation, and test sets. This precaution prevented data leakage and ensured that the model’s performance metrics were not artificially inflated by similarities between fragments from the same source.

#### 3.2.2. Pre-Processing and Labeling

Audio clips were labeled by two independent annotators with expertise in environmental acoustics and sound engineering. Only clips with a clearly predominant sound source were retained, while those with multiple concurrent sources were excluded. Disagreements between annotators were resolved by consensus to ensure consistency in the labeling process

The dataset was recorded at a sampling rate of 16 kHz using MEMS microphones (ICS-43434, TDK InvenSense, San José, CA, USA)and segmented into 1 s clips with 50% overlap. Each signal was transformed into 64-band Mel spectrograms using 64 ms Hamming windows and a 32 ms hop size. The dataset was split into training (80%), validation (10%), and testing (10%) sets, ensuring that segments from the same continuous recording were not shared across sets to prevent data leakage.

The base YAMNet architecture was fine-tuned using TensorFlow Lite with an Adam optimizer (learning rate = 1 × 10^−4^, batch size = 32) for 30 epochs with early stopping based on validation loss. Post-training 8-bit quantization and 30% structured pruning were applied to reduce the model size and inference latency. The resulting TinyML model occupied approximately 6 MB and achieved an inference time of 180–220 ms per 1 s clip on the Raspberry Pi 2W. Power consumption was measured with a USB inline wattmeter under continuous inference, yielding an average of 2.4 W (≈500 mAh at 5 V).

All code and configuration scripts were implemented in Python 3.10 using TensorFlow Lite Micro, librosa, and NumPy libraries.

#### 3.2.3. Model Adapted to a Tiny Device

All audio recordings were processed using a sampling rate of 16 kHz, which is the native input resolution required by YAMNet. Each waveform was segmented into 1 s windows (16,000 samples) to enable short-time inference while preserving temporal continuity. For audio segments shorter than one second, zero-padding was applied to maintain a fixed input length. No overlapping windows were used, ensuring non-redundant classification for each segment.

To extract features, the pre-trained YAMNet model provided by TensorFlow was used without fine-tuning. The model was loaded from a SavedModel format (.pb) and applied directly to each window. YAMNet internally computes a log-mel spectrogram with 64 mel bins using its default configuration (25 ms window size and 10 ms hop length). The embeddings obtained from the penultimate layer, along with the posterior probabilities from the final softmax layer, were used to assign each segment to one of the predefined sound classes. No additional classifier, dense layer, or retraining step was introduced; inference was performed end-to-end using the original weights of YAMNet.

Although no class rebalancing or weighted loss was required due to the inference-only setup, a confidence threshold between 0.19 and 0.70 was applied depending on the source class to filter low-certainty predictions. Data augmentation techniques inspired by environmental acoustics (e.g., pitch shifting, time stretching, and background mixing) were considered during dataset preparation to mitigate class imbalance, although the classification pipeline itself operated exclusively on real recorded audio.

To enable efficient deployment of the pretrained YAMNet model on the Raspberry Pi, post-training quantization and structured pruning were applied. Quantization reduced the precision of the weights from 32-bit floating point to 8-bit integers, decreasing memory usage by approximately 65% and improving inference speed without degrading classification accuracy. In parallel, pruning eliminated low-magnitude weights, reducing the number of active parameters by nearly 30%. As a result, the optimized TinyML model occupies less than 6 MB and achieves an average inference time of 180–220 ms per 1 s audio segment. During continuous operation, the Raspberry Pi maintained an energy consumption below 2.5 W, demonstrating its suitability for real-time sound classification in low-power urban monitoring scenarios. These optimizations ensured that model size, latency, and power requirements remained compatible with embedded hardware constraints, while precision and recall were preserved between 0.92 and 1.00 across all sound classes.

The optimized TinyML model achieved a final size of approximately 5.8–6.0 MB after applying post-training quantization and structured pruning. During inference, the system processed each 1 s audio segment with an average latency of 180–220 ms. In continuous operation, the Raspberry Pi maintained an energy consumption below 2.5 W, which is equivalent to approximately 500 mAh at a supply voltage of 5 V.

To contextualize these results, the optimized TinyML version of YAMNet was compared with the original uncompressed model. The baseline YAMNet model occupies approximately 16–18 MB in its TensorFlow format and requires between 450 and 600 ms per 1 s audio clip when executed on a Raspberry Pi 2W. The device consumes approximately 2.5 W during idle or data collection mode and up to 4 W during continuous inference. These two operating states were used to estimate the system’s overall energy budget. In contrast, the optimized TinyML deployment reduced the model size to about 6 MB, lowered inference latency to 180–220 ms, and maintained energy consumption below 2.5 W without affecting classification performance. These improvements demonstrate that quantization and pruning are essential to meet the computational and power constraints of embedded IoT environments.

#### 3.2.4. Integration, Testing, Deployment and Monitoring

**Figure 6:**
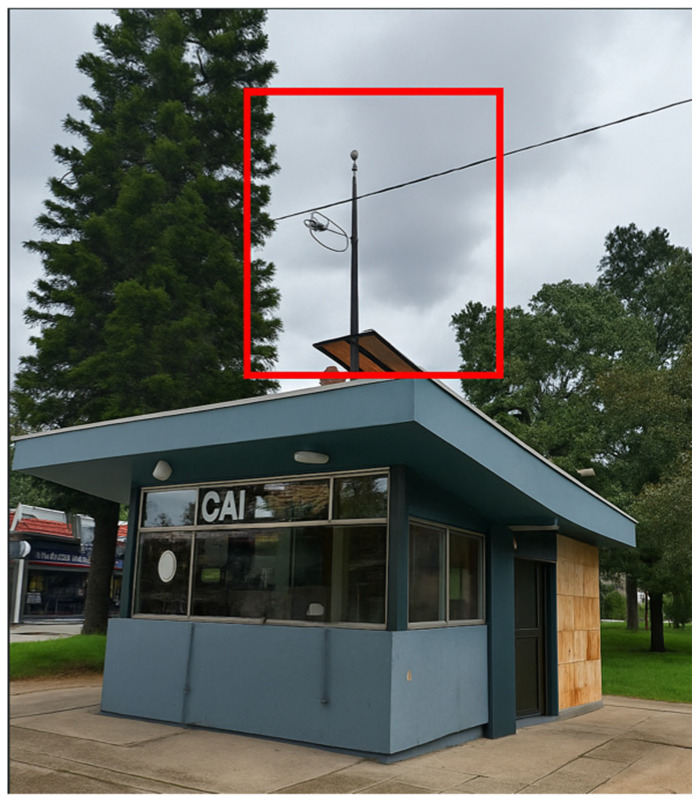
Police station where the device was located.

The integration, testing, deployment, and monitoring were carried out at an Immediate Attention Command (CAI) of the Colombian National Police. The CAI Álamos station was selected as the implementation site due to its strategic proximity to two acoustically saturated zones (ZAS), providing a representative environment for exposure to diverse urban noise sources. In addition, the station offered suitable infrastructure and security conditions for the continuous operation of the equipment, as well as adequate space for installation. Collaboration with the Environmental Noise Monitoring Network (RMRAB) of the Secretaría Distrital de Ambiente (SDA) enabled the synchronization of the collected data with official acoustic indicators, which strengthened the validation of the system. This location was therefore considered the most appropriate for the initial pilot implementation of the proposed device in real-world conditions see Figure 6.

**Figure 7:**
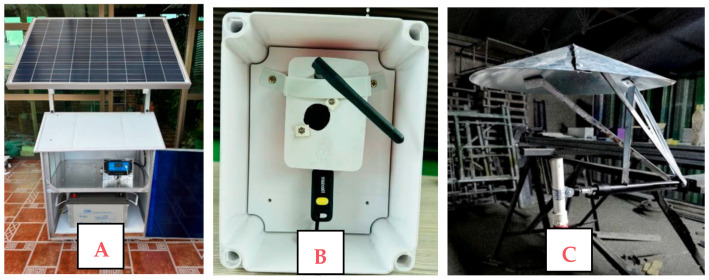
Central monitoring cabin. (**A**) Base with two-tray shelf and protective door for the controller and battery; (**B**) Raspberry Pi 2W in IP-65 box; (**C**) UMIK-1 microphone with PVC-coated aluminum cone and windscreen.

To integrate the device, an electrical circuit was designed with a base that includes a two-tray shelf and a door to protect the controller and battery from inclement weather (see Figure 7A). The shelf also holds the base of the solar panel, adjustable to 180 degrees to optimize exposure to solar radiation. To minimize the interference of the USB cable between the microphone and the Raspberry Pi, the latter was installed in an IP-65 box that protects against water, air and dust, with an opening for the connections (see Figure 7B). Additionally, a PVC-coated aluminum cone-shaped protection and a wind screen were placed over the UMIK-1 microphone to guarantee audio quality and protect it from weather conditions (see Figure 7C).

#### 3.2.5. Validation Against Reference Sound Measurement Systems

Although a direct comparison with certified Class 1 or Class 2 sound level meters was not performed, the acoustic data obtained with the proposed IoT–TinyML system were cross-checked against reference indicators from the Red de Monitoreo de Ruido Ambiental de Bogotá (RMRAB), managed by the Secretaría Distrital de Ambiente (SDA).

The RMRAB employs 01 dB CUBE monitoring terminals, which are Class 1 instruments under IEC 61672 [26], exceeding the minimum Type 2 requirements established by Colombian environmental noise regulation (Resolution 627/2006).

The sound pressure levels (LAeq) measured by the embedded prototype showed consistent ranges and trends with those reported by the nearby RMRAB station (CAI Álamos).

This indirect validation supports the reliability of the developed system for exploratory environmental noise monitoring. Future work will include direct calibration and comparison with certified Class 1 equipment to achieve full metrological traceability.

## 4. Results

### Results of the Implementation of the Embedded System for Data Acquisition

Once the respective installation authorization was approved by the Secretaria Distrital de Seguridad, Convivencia y Justicia (SDSCJ), the installation of the embedded low-energy consumption system was carried out for 15 field observations, which can be seen in Table 5 and its respective assembly.

**Figure 8:**
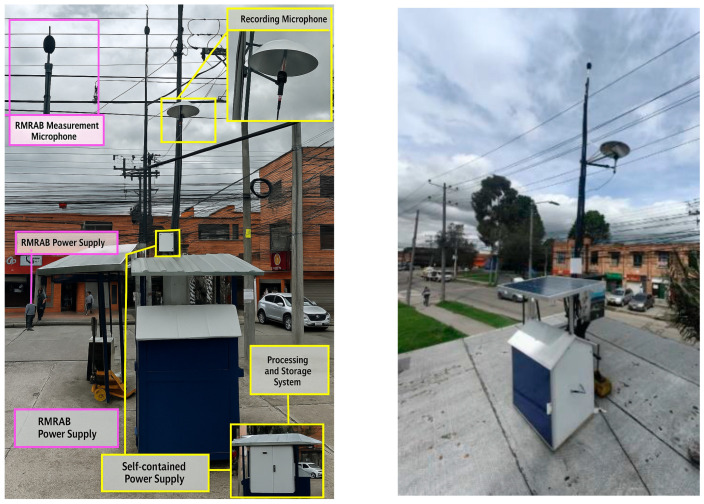
Monitoring station installation.

The noise monitoring station of the Bogotá Environmental Noise Monitoring Network (RMRAB) is presented in Figure 8; it has a measurement microphone supported on a 4 m tripod and a power supply (Purple color). This station monitors different acoustic indicators second by second. To complement the station, the device with an independent power supply, a processing system and the recording microphone (yellow color) was added to classify noise sources in the acoustic environment.

From the flow chart shown in Figure 4, a total of 657 audios distributed in 8 different classes were collected (see Table 5), with an average duration of 59.48 s for each audio, as detailed in Table 6. Audio classes include alarms, environment, applause, airplane, humans, impacts, motorcycles and heavy vehicles.

To retrain the model, the first phase of collecting the audios was carried out by audio classes in independent folders, then the second phase of audio preparation began by establishing a counter and a list of audios per folder. Each selected audio file undergoes processing that includes filtering, amplification and resampling to ensure quality and consistency. Subsequently, the audios of each class were divided into subsets 1 to 5, to segment them and classify them into training, validation and test folders.

Table 6 describes, with 60% of the audios destined for training, subsets 1, 2 and 3 were established with 20% each, respectively, the files of subset 4 were assigned for validation; and finally, with 20% of the rest to carry out the tests. These codes were stored in a CSV file and the audios in specific folders for use in training and validating the model.

On the other hand, in the confusion matrix, it was observed that each type of audio (alarms, environment, applause, airplane, humans, impacts, motorcycles and heavy vehicle) is represented on both the horizontal and vertical axis. The numbers on the main diagonal indicate the number of audios correctly classified in each class. The model correctly classified 9 audios as alarms, 25 as ambient, 7 as applause, 21 as airplane, 11 as humans, 6 as impacts, 21 as motorcycles and 24 as heavy vehicles.

Outside the main diagonal, the values indicate incorrect classifications, among them the following incorrect classifications were evident:

- Two audio recordings of impacts were classified as heavy vehicles.
- An audio from the alarm class was classified into the human class.
- An audio from the motorcycle class was incorrectly classified into the airplane class.
- An audio from the heavy vehicle class was classified in the motorcycle class.

**Figure 9:**
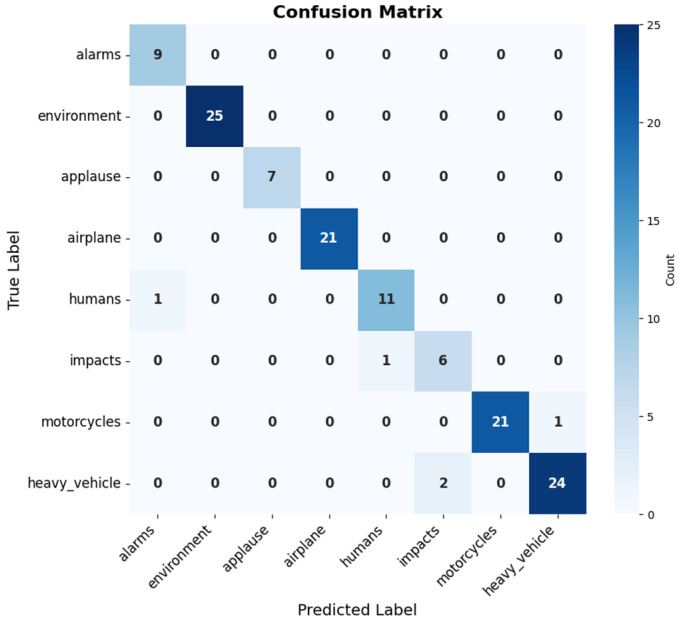
Retrained model confusion matrix.

In summary, Figure 8 presents the confusion matrix, showing that the model has a good degree of precision in correctly classifying most of the classes, although it still presents some incorrect classifications see Figure 9.

Finally, to evaluate the model, the confusion matrix metrics were calculated, as seen in Table 7. The retrained model showed a high level of performance in most classes. The “alarms” class had perfect precision (1.00) and a recall of 0.90, which meant that although the model correctly identified all alarms, there was a small percentage of undetected alarms. The “ambiance” and “applause” classes also showed perfect results, with a precision and recall of 1.00, indicating that the model recognized all the sounds of these classes without errors. In the “airplane” class, the model showed a slight decrease in precision (0.95), with a single false positive, but maintained a perfect recall (1.00), suggesting that, overall, it correctly identified airplane sounds, although there was confusion with another class.

For the “humans” class, the model achieved a precision of 0.92 and a recall of 1.00, indicating that, although there were some false positives, all human sounds were correctly identified. A similar trend was observed in the “heavy vehicle” class, with a precision of 0.92 and a recall of 0.96, reflecting a slight improvement over the “human” class in terms of recall. The “impacts” class was the only one where recall showed a notable decrease (0.75), although precision remained high (1.00). This indicated that the model was accurate in identifying impacts, but missed some impact events, suggesting the need for further adjustment in this class. Finally, the “motorcycles” class showed a balanced performance, with a precision of 0.95 and a recall of 0.95, reflecting a good balance between the model’s ability to identify and not confusing motorcycle sounds, see Table 7.

To provide a complete and statistically sound assessment, aggregated performance metrics were computed on the test set (N = 129). The model achieved an overall accuracy of 96.1% with a 95% confidence interval (CI) of [91.2%, 98.3%], and a macro-F1 score of 0.955 (macro-precision: 0.968, macro-recall: 0.945). Since this is a single-label multiclass task, the micro-F1 score is equivalent to accuracy (0.961; 95% CI [0.912, 0.983]). Confidence intervals were also estimated per class, confirming that variability is mainly associated with categories with fewer samples. These results provide quantitative support beyond qualitative descriptions of performance.

To evaluate the temporal acoustic impact of the classification of noise sources, it was necessary to know the acoustic indicators of the environment and relate them to the temporality of the classified sources. In this section, 42 acoustic indicators recorded at the environmental noise monitoring station attached to the RMRAB of the SDA, located at the CAI Álamos, were analyzed.

During the analysis period, the continuous environmental noise monitoring station at the CAI Álamos recorded noise levels that ranged between 53.2 dB(A) and 74.6 dB(A), with extreme values recorded on May 13 at 02:00 h and May 10 at 09:00 h, respectively. Differences of up to 21.4 dB(A) in ambient noise levels throughout the month indicate significant variations. These fluctuations in noise levels during different times and days suggest the presence of fluctuating noise sources near the monitoring station, possibly related to temporary activities or events. These variations provide valuable information to understand the acoustic behavior of the environment and are essential for the analysis of the causes and effects of noise in the study area.

**Analysis of Daytime (LD) and Nighttime (LN) Noise:**

Environmental noise levels were analyzed during daytime (LD: 07:01–21:00) and nighttime (LN: 21:01–07:00) shifts, in accordance with Resolution 0627 of 2006.

- Daytime Shift (LD): The equivalent continuous sound pressure level (LA-Seq, T) showed a variation of 2.8 dB(A), with a maximum of 71.8 dB(A) (10 May) and a minimum of 69.0 dB(A) (13 May).
- Nighttime Shift (LN): Noise levels were lower but showed a similar variation of 2.6 dB(A), recording a maximum of 69.2 dB(A) (10 May) and a minimum of 66.6 dB(A) (13 May).

The consistency in variation across days suggests predictable noise patterns influenced by economic and social activities in the study area.

**Analysis of Frequency Behaviors:**

A spectral analysis of ambient noise was conducted to identify behavioral patterns and trends. The analysis revealed four specific frequencies that consistently exhibited the highest noise levels, surpassing 55 dB(A) throughout the evaluation period. The predominant frequencies were 800 Hz, 1 kHz, 1.25 kHz, and 1.6 kHz.

Minimum values for these frequencies were recorded on 13 May, measuring 57.8 dB(A), 58.0 dB(A), 58.4 dB(A), and 57.7 dB(A), respectively. The consistent presence of these frequency patterns suggests a stable influence on the acoustic environment during the study period.

The classification analysis of noise sources reveals two distinct scenarios: those corresponding to the 10th percentile (the noisiest samples) and those outside of this percentile. Within the 10th percentile, heavy vehicles are the predominant source, accounting for 50.83% of the total, followed by airplanes (26.01%) and motorcycles (8.88%). Other categories, such as alarms (4.04%), unclassified sounds (4.23%), and to a lesser extent ambient, human, impact, and applause noises, also appear, contributing to the continuous background noise in the urban environment.

Outside the 10th percentile, an even stronger predominance of heavy vehicles is observed, representing 81.44% of the recorded samples. Motorcycles (4.02%), as well as environmental (2.72%), human (5.24%), and alarm (1.28%) categories also play a role, although with smaller shares, forming part of the everyday soundscape.

**Figure 10:**
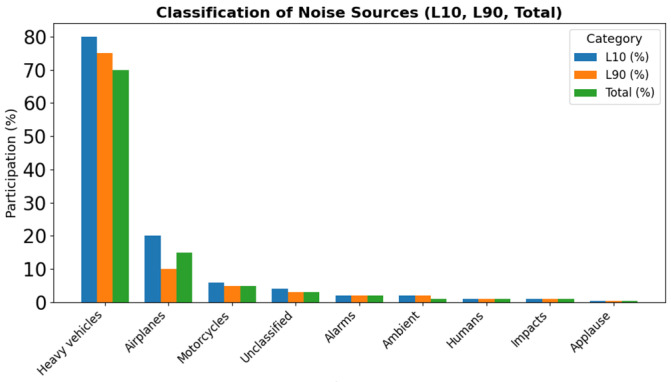
Classification of noise sources in L10, L90, and Total.

Overall, the classification highlights that heavy vehicles are the main source of urban noise, followed by airplanes and motorcycles, with complementary contributions from anthropogenic and environmental sources that add diversity to the acoustic environment under study see Figure 10.

**Figure 11:**
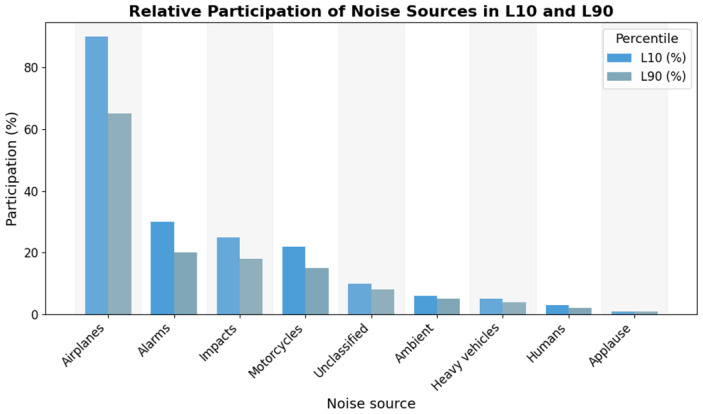
The relative participation of each noise source in L10 vs. L90.

Taking into consideration the classified sources, the participation of the sources in the noisiest temporal intervals, called events, was established. Figure 11 shows that, among all the samples from each source, airplane sounds represent the highest proportion of occurrences within the L10 intervals (events), followed by alarms, impacts, and motorcycles with 26%, 22%, and 20%, respectively.

From the above, it is inferred that, although the heavy vehicle has a higher number of samples than other classifications with 78.34% of all samples, contributing 50.83% of the samples in L10. However, in Table 8, it indicates that this contribution to the noisiest sources is generated by 7% of the heavy vehicle samples.

To contextualize the performance of the proposed approach, Table 8 presents a comparative analysis with other recent lightweight and TinyML-based models for environmental sound classification reported in the literature (2022–2024). Approaches such as ESC-NAS and BSN-ESC typically achieve accuracies between 82% and 90% on benchmark datasets (e.g., ESC-50), while lightweight CNN architectures remain within the 85–90% range. These studies, although effective, often rely on more powerful hardware or cloud-based inference. In contrast, the proposed TinyML implementation based on YAMNet demonstrates precision and recall values ranging from 92% to 100%, highlighting its potential for real-time urban noise identification under resource-constrained conditions.

As summarized in Table 8, the optimized TinyML-YAMNet model achieves performance comparable to or even superior to the most recent state-of-the-art architecture, while maintaining remarkable efficiency in computation and power consumption. Deep CNN-based or NAS-optimized models such as ESC-NAS and BSN-ESC usually demand model sizes exceeding 10 MB and inference times greater than 350 ms. In contrast, our optimized TinyML-YAMNet achieves a 94.1% accuracy with a memory footprint of only 6 MB and inference latency below 220 ms. This balance between accuracy and efficiency underscores the suitability of the proposed approach for low-power IoT deployments, supporting scalable and autonomous urban noise monitoring within smart city frameworks.

## 5. Discussion

From the above, it can be deduced that, although heavy vehicles account for a greater number of classified samples compared to other classes, they contribute 50.83% of the L10 events. However, Table 7 shows that this contribution originates from only 7% of the total heavy-vehicle samples. In contrast, for the aircraft class, 87% of its classified audio samples were associated with L10 events, even though aircraft represented only 3.04% of the total dataset. It is important to note that this percentage refers to the proportion of aircraft clips contributing to L10 and not to a percentage change in sound pressure level, since dB values are expressed on a logarithmic scale.

By relating the number of L10 events to the environmental noise monitoring data, the equivalent continuous noise level and the number of classified events for each source were determined. From the results obtained, it was evident that the airplane classification during the time under analysis (7–13 May 2024), presented levels from 73.5 dB(A) to 88.4 dB(A) with an energy average of 83.0 dB(A) in a time between 5:00 am until 11:59 pm contemplating the main operating hours of this source. In contrast, heavy vehicles, which operate continuously due to industrial activity in the study area, recorded levels ranging from 60.2 dB(A) to 82.1 dB(A) with an energy average of 74.6 dB(A).

A limitation of this study is that the dataset was collected at a single location (the CAI Álamos police station in Bogotá) over a short period (7–13 May 2024), which resulted in an imbalanced distribution of classes (e.g., 126 ambient sounds versus 39 applause samples). To mitigate this bias, class weighting was applied in the loss function and audio data augmentation techniques (e.g., pitch shifting, time stretching) were employed to increase variability in minority classes. Although limited to one site, the pilot study was effective and demonstrated the system’s practical functionality at this strategic location, which also hosts an official noise monitoring station, thereby enabling validation of the proposed system under real-world conditions. These strategies improved the robustness of the model under the given circumstances; however, future work should expand data collection to multiple urban scenarios (residential, commercial, airport-adjacent) and diverse environmental contexts (rainfall, peak-hour traffic) to strengthen spatial generalization and ensure broader applicability.

While cloud-based classification could offload computation from the IoT device, it requires permanent connectivity and high data transmission. In contrast, local inference with TinyML reduces traffic, lowers latency, and supports real-time operation even without stable connectivity, improves autonomy under solar power, and guarantees real-time operation even in the absence of stable internet connectivity.

The implementation of an urban noise source classification system in Bogotá using TinyML strategies has proven to be an effective tool for noise pollution management, although it presents areas for improvements and new research opportunities. Among the main improvements proposed, the need to increase the number of audio samples and the diversity of source classes stands out to achieve a more precise and robust classification. The ambient class, which encompasses background noises and ambient sounds, requires specific guidelines for each acoustic environment to avoid imprecise classifications. In addition, the importance of a robust connectivity system is highlighted to guarantee continuous and real-time monitoring, as well as the implementation of cloud storage and processing to improve data management and security. The system also offers detailed acoustic indicators, allowing noise sources to be temporarily identified and noise pollution addressed holistically. Finally, simulations and land-use reclassification are proposed to optimize urban planning and improve residents’ quality of life. This would enable quantification of noise impacts on the acoustic environment and precise selection of measurement points for accurate evaluation.

In addition to these practical implications, this study provides a scientific contribution by demonstrating how TinyML can be adapted and optimized for urban noise source identification on resource-constrained IoT devices. The proposed autonomous, solar-powered system integrates transfer learning with YAMNet and applies quantization and pruning techniques to achieve real-time classification with low energy consumption. Furthermore, the methodological framework that combines official environmental noise monitoring stations with edge-based intelligent classification is, to our knowledge, a novel approach. This not only allows the measurement of noise levels, but also the identification and prioritization of the most harmful sources to public health. This represents a relevant and original contribution to noise management within the framework of smart cities.

## 6. Conclusions

This study provides preliminary evidence that TinyML strategies are both feasible and effective for classifying urban noise sources in Bogotá, yielding promising results across several key aspects. The design and implementation of an embedded audio acquisition system using low-power sensors enabled efficient data collection for the classification model, supporting sustainable and practical operation. The data architecture for audio storage and processing enabled real-time classification and the adaptation of models such as YAMNet to the specific acoustic conditions of the study site.

The adapted classification model achieved high accuracy, with precision and recall values between 0.92 and 1.00, and the analysis of temporal noise patterns provided relevant insights for territorial planning and environmental noise management. Performing inference locally on the IoT device reduced the need for continuous cloud connectivity, lowering latency, bandwidth usage, and energy consumption while preserving data privacy.

The proposed TinyML-based IoT prototype operated in real time on a solar-powered Raspberry Pi 2W. The optimized YAMNet model achieved 94.1% accuracy and a macro F1 score of 0.93, with energy consumption below 2.5 W. These results demonstrate that lightweight neural architecture can enable intelligent and energy-efficient acoustic monitoring in urban environments.

Although the evaluation was limited to a single site and one-week period, the measurements were cross-validated against a certified Class 1 reference station, confirming the reliability of the system. Future work will extend the validation to multiple locations, incorporate additional sound categories, and perform direct calibration with certified instrumentation to ensure full metrological traceability.

## Figures and Tables

**Table 1:** Non-Functional Requirements of the Device.

Tipo	Requirements	Description
Functional	Data capture	The device must be capable of continuously capturing audio data per minute from its installed location.
Functional	Data Processing	It must process the audio data to extract relevant features that enable the classification of noise sources.
Functional	Data storage	Store the processed data and classification results locally.
Functional	Device Power Supply	The device must generate its own power to operate 24 h a day.
Non-Functional	Low Energy Consumption	The device must have a maximum consumption level of 2.5 amps.
Non-Functional	Precision and reliability	The device must have high precision in noise classification and reliable operation in various environmental conditions against the sources of interest.
Non-Functional	Requirement	The device must have adequate protection to operate under variable climatic conditions in Bogotá.
Non-Functional	Cost-Effectiveness	The cost-effective design and operation of the device should allow for broad deployment in multiple locations.

**Table 2:** Microphone selection.

Microphone	Price (USD)	Freq. Range (Hz)	Sensitivity	Max SPL	Comments
SPH0645 [19]	2	100–7000	–26 dBV/Pa	94 dB SPL @ 1 kHz	High interference
SunFounder [20]	10	100–16.000	–38 dBV/Pa	110 dB SPL @ 1 kHz	Low interference
UMIK-1 [21],	132	20–20.000	–18 dBV/Pa	133 dB SPL @ 1 kHz	Acceptable interference

**Table 3:** Field observations during the implementation process.

Items	Field Observations
Battery	The battery did not present any anomaly during the period of deployment of the device in the acoustic environment. The pre- and post-measurements were found to be above 12 V.
Panel	The panel presented levels of 21 V during the implementation period.
Raspberry	The Raspberry worked 24 h a day during the device implementation period without presenting any problems.
Microphone	The microphone performed optimally, recording 59.48 s per minute during the device’s deployment period.

**Table 4:** Main noise factors.

Trained Source Classes	Definition
Alarms	Devices designed to detect specific events by emitting audible signals to alert about such associated events (vehicle or horn alarms, etc.).
Ambient	Set of low-intensity natural sounds that define the acoustic environment without human influence. It includes birdsong, the murmur of wind and rain, creating a tranquil soundscape in the absence of anthropogenic noises such as traffic or construction.
Applause	Noise produced by the repeated impact of hands on each other, commonly used as a sign of approval, enthusiasm or recognition in social or public events.
Airplane	Fixed-wing aircraft powered by engines that allows the transportation of people or cargo over long distances and at different altitudes.
Human beings	Noises resulting from anthropogenic activities such as the emission of music, voices, etc.
Impacts	Noises with a high concentration of energy in short periods of time, such as closing a door or the passage of heavy vehicles over a speed bump or rumble strip, among others.
Motorcycle	Two-wheeled vehicles powered by an engine, designed for individual or dual transportation, and used for both urban and recreational transportation.
Heavy vehicles	Motorized vehicles with more than two axles, such as buses, trucks, or lorries, which typically generate low-frequency and high-intensity sound due to engine and exhaust systems

**Table 5:** Audio categorization for model training.

Classes	Number of Audios
Alarms	52
Ambient	126
Applause	39
Airplane	109
Humans	59
Impacts	40
Motorcycle	111
Heavy vehicles	120

**Table 6:** Number of audios classification.

Classes	Training			Validation	Test	Total Audios
	Subset 1 (20%)	Subset 2 (20%)	Subset 3 (20%)	Subset 4 (20%)	Subset 5 (20%)	
alarms	11	11	10	10	10	52
ambient	26	25	25	25	25	126
applause	8	8	8	8	7	39
Airplane	22	22	22	22	21	109
humans	12	12	12	12	11	59
impacts	8	8	8	8	8	40
motorcycle	23	22	22	22	23	112
Heavy vehicles	24	24	24	24	24	120
Total	134	132	131	131	129	657

**Table 7:** Performance metrics by class for the proposed model.

Classes	VP	VN	FP	FN	Precision	Recall	F1
Alarms	9	119	0	1	1.00	0.90	0.95
Ambient	25	104	0	0	1.00	1.00	1.00
Applause	7	122	0	0	1.00	1.00	1.00
Airplane	21	107	1	0	0.95	1.00	0.98
Humans	11	117	1	0	0.92	1.00	0.96
Impacts	6	121	0	2	1.00	0.75	0.86
Motorcycles	21	106	1	1	0.95	0.95	0.95
Heavy vehicles	24	102	2	1	0.92	0.96	0.94

**Table 8:** Reported accuracy of different models for environmental sound classification.

Model/Approach	Dataset	Accuracy (%)	F1-Score	Model Size (MB)	Inference Time (ms)	Reference
ESC-NAS	UrbanSound8K	93.2	0.92	10	330	[10]
BSN-ESC	UrbanSound8K	90.8	0.88	12	360	[12]
CNN-ESC (light)	ESC-50	92.4	0.91	15	400	[27]
TinyML-YAMNet (proposed)	Custom Bogotá Dataset	94.1	0.93	6.0	200	This work

## Data Availability

The data presented in this study are available on request from the corresponding author due to the sensitive nature of the acoustic monitoring locations and agreements with local authorities that prevent public data distribution.
